# Emerging Technologies in Edge Computing and Networking

**DOI:** 10.3390/s24041271

**Published:** 2024-02-17

**Authors:** Javier Prieto, Ramón J. Durán Barroso

**Affiliations:** 1 BISITE Research Group, University of Salamanca, 37007 Salamanca, Spain; 2 Optical Communications Group, Universidad de Valladolid, 47011 Valladolid, Spain

## 1. Introduction

The global evolution of the Internet is experiencing a notable and inevitable change towards a convergent scenario known as the Internet of Things (IoT), where a large number of devices with heterogeneous characteristics and requirements have to be interconnected to serve different verticals, such as smart cities, intelligent transportation systems, smart grids, (ITS) or e-health [1,2,3,4,5,6]. In order to satisfy the strict requirements related to end-to-end latency and the scalability of new services, as well as the processing of the massive volume of data that they generate, it is necessary to design and deploy a decentralized service platform architecture based on MEC (multi-access edge computing) and/or fog computing, with the aim of reducing the distance between the source of data and the processing resources. Moreover, 5G, and especially 6G, have been proposed to fulfil the strict latency and scalability constraints, providing cell-less multiaccess networks controlled by efficient artificial intelligence techniques and supported by MEC.

All these elements are forcing a holistic redesign of communication networks and distributed computing and storage architectures to maximize the performance/cost ratio, meeting the demanding challenges of latency, scalability, bandwidth, availability and flexibility that future services will require. This evolution is poised to influence all network segments, including wireless, optical, IP, etc. It hinges on the seamless integration of SDN, NFV, and the slicing paradigm, facilitated by the utilization of AI [7,8,9,10,11,12,13,14,15,16,17,18,19,20,21,22,23].

However, such distributed and heterogeneous systems can suffer from attacks that can affect the integrity of infrastructure, applications, users, and data. necessitating a comprehensive understanding of the cybersecurity problem, encompassing various levels [24,25,26,27,28,29].

This Special Issue has been developed in the context of the H2020 IoTalentum project. It presents original research articles and communications from researchers in the edge computing and networking communities, exploring emerging technologies that enable the secure integration of edge computing and networks.

## 2. Overview of Contribution

The Special Issue titled “Emerging Technologies in Edge Computing and Networking” comprises eleven high-quality papers, including one Communication and ten research articles covering cutting-edge topics such as new edge computing architectures, network virtualization, edge node design (hardware/software), or compression of deep neural networks for edge devices.

Nakazato et al. (contribution 1) design a fully virtualized architecture for Multi-Access Edge Computing (MEC) 5G cellular networks, aimed for local use cases (e.g., stadiums and campuses). In this sense, the authors propose a MEC/Cloud Orchestrator that aims to intelligently select deployment options, and they assess its performance through a Beyond 5G testbed, demonstrating reduced latency and more stable throughput compared to traditional cloud services.

Cao et al. (contribution 2) present a fast and energy-efficient vehicular edge computing framework for optimal offloading decisions in urban scenarios with overlapping Roadside Units (RSU) coverage that minimize delay and energy consumption. The proposed multi-objective offloading method employs an improved non-dominated sorting genetic algorithm-II algorithm and introduces a QoS model to find the optimal offloading strategy with low complexity.

Maia et al. (contribution 3) describe an integrated platform that simulates and monitors realistic industrial conditions in a digital twin-based three-tier architecture: edge, platform, and enterprise tiers. The designed platform can be integrated with the ERP system and captures, analyzes, and correlates a wide range of events, such as attacks, vulnerabilities or safety issues that are tracked by sensors and systems in various domains of the factory.

Bruns et al. (contribution 4) propose a mobile crowdsourcing system that can improve the task assignment and respond to failure-causing events by implementing different mechanisms for outcome prediction and task coordination. The proposed task assignment mechanisms rely on an opportunistic approach and on a market-based model and could be used as a basis for task transfers among the edge nodes in edge computing architectures.

Algarvio et al. (contribution 5) describe a strategic bidding process for retailers to submit bids to a wholesale market composed by a day-ahead market, an intraday market, and a balancing market. This work aims to consider prosumers as part of the portfolio of retailers, where distributed edge computing resources for demand and price forecast may play a pivotal role.

Chen et al. (contribution 6) investigate the load imbalance caused by the use of function aggregation in the deployment of service function chaining (SFC) in the air–ground network. The authors propose a virtual network function (VNF) aggregation scheme based on task similarity that obtains the most suitable physical nodes according to various attributes of physical network nodes and demonstrate the performance by simulations with a network topology of 25 points and 45 edges.

Marco-Detchart et al. (contribution 7) design a low-cost edge-AI device that incorporates the necessary hardware and software components for automatically detecting plant diseases from a set of images of a plant leaf. The authors use edge technology to capture multiple images of the leaves and implement data fusion techniques to enhance the classification process and improve its robustness, avoiding the transmission of images over the network.

Hafid et al. (contribution 8) explores sharding-based Proof-of-Stake (PoS) Blockchain protocols, focusing on their key components and delving into the consensus mechanisms of PoS and practical Byzantine Fault Tolerance (pBFT). In this context, edge computing offers the lowest latency and cost for computing delivery and consumption and is particularly well-suited for PoS deployments due to the higher networking requirements imposed by the Proof-of-Work (PoW) consensus.

Choi et al. (contribution 9) describe a method to simplify and quantify a deep neural network (DNN) used for object detection with the aim to embed it into a real-time edge device. The authors analyze five channel pruning methods for network simplification and two parameter quantization methods for network quantization and demonstrate that the detector can operate in real-time embedded onto a System-on-Chip (SoC) that integrates CPU, GPU, and DSP for high-performance Internet of Things (IoT).

Leng et al. (contribution 10) propose an edge system for license-plate-recognition based on semisupervised learning (SSL) suitable for tiny edge gateways that overcomes inherent limitations such as high latency and energy consumption (throughout pruned feature extraction network and compressed feature fusion network). The authors use a hybrid edge/cloud computing approach, where cloud computing is used to upload data gathered at the network’s edge and continually update the overall neural network model.

Finally, Tominaga et al. (contribution 11) provide in their Communication new improvements for image generation from text descriptions based on stacked generative adversarial networks (StackGAN). The proposed method aims to suppress mode collapse and to reduce the variations in the generated images, which is crucial to reduce computation and communication overhead in future edge computing-based and federated generative adversarial networks (GAN).

## 3. Conclusions

This Special Issue has collected a set of significant and visionary contributions addressing challenges in edge computing and networking technologies. The presented articles and communications have proposed not only new edge computing architectures, but algo new designs of edge nodes as well as compression methods that enable researchers to embed demanding deep neural networks into tiny edge devices. Finally, the Special Issue has also comprised a set of application papers in sectors such as energy, blockchain and vehicular networks where edge computing reaches a lower latency and computational cost than traditional cloud-based approaches.
